# Obituary, Dr. Saleh Zahedi-Asl

**DOI:** 10.5812/ijem.18719

**Published:** 2014-04-01

**Authors:** Fereidoun Azizi, Azita Zadeh-Vakili, Niloofar Shiva

**Affiliations:** 1 Endocrine Research Center, Research Institute for Endocrine Sciences, Shahid Beheshti University of Medical Sciences, Tehran, IR Iran; 2 Cellular and Molecular Research Center, Obesity Research Center, Research Institute for Endocrine Sciences, Shahid Beheshti University of Medical Sciences, Tehran, IR Iran

A highly respected, renowned academician of his generation, Dr. Saleh Zahedi-Asl passed away on the 3rd November 2013, leaving behind a lasting legacy. His sudden, totally unexpected demise left us all shocked and bewildered, but forced to come to terms with the tragic loss. Dr. Zahedi was the Editor-in-Chief of both the International Journal of Endocrinology and Metabolism and the Iranian Journal of Endocrinology and Metabolism, and the Deputy Director of the Research Institute for Endocrine Sciences (RIES), Shahid Beheshti University of Medical Sciences, Tehran. In the past he had been the editor in-chief of the Scientific Medical Journal of Ahvaz University of Medical Sciences (AUMS). A member of several editorial boards, both international and national, research councils and national examination boards and societies, Dr. Zahedi was never intimidated by controversy, did not always concur with current trends and was always available to and willing to corroborate with and counsel authors within and outside Iran.

Born in Ardebil, Iran, on August 29th 1951, Dr. Zahedi graduated with an honors bachelor’s degree in basic sciences from the University of Tabriz (1973) and went on to obtain his master’s in physiology from the university of Jundishapur, Ahvaz (1978). Dr. Zahedi then proceeded to the University Of Newcastle Upon Tyne, UK for his Ph.D. (1981), during which he developed radioimmunoassay for insulin, two kinds of glucagon (pancreatic & gut) and GIP (glucose-dependent insulinotropic pepetide). He was granted a MRC postdoctoral fellowship to work with Dr. John Brown on projects involving raising monoclonal antibodies to hormones, extraction, purification and sequencing of new biologically active peptides. He had published over 108 papers in Persian and 81 in English and edited one book and translated four books from English to Farsi. Dr. Zahedi’s publications include many papers on both animal and human research, as well as some valuable papers in the field of endocrinology and gastro-intestinal physiology. He had recently worked on the developmental effects of thyroid hormones and demonstrated for the first time that maternal hypothyroidism could affect carbohydrate metabolism and insulin secretion in adult offspring in rats. He also had some other publications on laboratory methods including the development of modified RIA and ELISA methods. He used the assays mentioned to study the plasma pancreatic and gut glucagon, GIP, insulin and gastrin responses to feeding in controls and patients with duodenal ulcer (DU) disease, who were significantly hypoglycaemic before and after breakfast compared with controls, and had significantly higher insulin, GIP and gut glucagon responses after feeding, demonstrating that the relative hypoglycaemia was enough to activate the vagus nerves and to cause peptic ulcer; he further showed that secretin provocation tests, usually used to diagnose pancreatic gastrinoma, produce marked insulin release in patients with primary hyperparathyroidism but not in controls or in patients with gastrinoma or those with DU disease.

In another study, he reported that the gut hormone cholecystokinin (CCK) has a similar distribution in human brain compared with results documented for guinea pig and porcine brains. Using the gastrin assay and column chromatography to identity CCK molecules of different sizes, he also showed that in the cerebral cortex most of the CCK is in the grey matter and that in patients with Alzheimer disease there is significantly more glucagon in grey matter than in controls. He was very involved in teaching different aspects of human physiology, especially the GI tract, the endocrine system and circulation to both medical and post-graduate students, and had conducted well over fifty theses. To date he had conducted over 100 research projects, established a research laboratory and was currently working mainly on the behavior of isolated islets and isolated aorta from diabetic animals. He received many awards, including one for outstanding professor in 2011.

A workaholic, Dr. Zahedi was a member of the Iranian Academy of Medical Sciences. Apart from this he held almost thirty-five other important key positions; he was a member and chairman of the physiology and pharmacology boards of the country, and was a founding member of continuing education in Iran.

So many distinctive compliments come to mind when trying to eulogize Dr. Zahedi for what he was renowned and respected for, but space and words to describe him are lacking. Devout, self- effacing, affable, always the gracious gentleman, Dr. Zahedi is and will remain with us in our thoughts and prayers for a long long time.

